# A time- and dose-dependent STAT1 expression system

**DOI:** 10.1186/1472-6750-6-48

**Published:** 2006-12-21

**Authors:** Nicole R Leitner, Birgit Strobl, Marion Bokor, Ronald Painz, Thomas Kolbe, Thomas Rülicke, Mathias Müller, Marina Karaghiosoff

**Affiliations:** 1 Institute of Animal Breeding and Genetics, Veterinary University of Vienna, Veterinärplatz 1, 1210 Vienna, Austria; 2 University Center Biomodels Austria, Veterinary University of Vienna, Veterinärplatz 1, 1210 Vienna, Austria; 3 Department of Agrobiotechnology, IFA-Tulln, Institute of Biotechnology in Animal Production, University of Natural Resources and Applied Life Sciences, 3430 Tulln, Austria; 4 Institute of Laboratory Animal Sciences, Veterinary University of Vienna, Veterinärplatz 1, 1210 Vienna, Austria

## Abstract

**Background:**

The signal transducer and activator of transcription (STAT) family of transcription factors mediates a variety of cytokine dependent gene regulations. STAT1 has been mainly characterized by its role in interferon (IFN) type I and II signaling and STAT1 deficiency leads to high susceptibility to several pathogens. For fine-tuned analysis of STAT1 function we established a dimerizer-inducible system for STAT1 expression *in vitro *and *in vivo*.

**Results:**

The functionality of the dimerizer-induced STAT1 system is demonstrated *in vitro *in mouse embryonic fibroblasts and embryonic stem cells. We show that this two-vector based system is highly inducible and does not show any STAT1 expression in the absence of the inducer. Reconstitution of STAT1 deficient cells with inducible STAT1 restores IFNγ-mediated gene induction, antiviral responses and STAT1 activation remains dependent on cytokine stimulation. STAT1 expression is induced rapidly upon addition of dimerizer and expression levels can be regulated in a dose-dependent manner. Furthermore we show that in transgenic mice STAT1 can be induced upon stimulation with the dimerizer, although only at low levels.

**Conclusion:**

These results prove that the dimerizer-induced system is a powerful tool for STAT1 analysis *in vitro *and provide evidence that the system is suitable for the use in transgenic mice. To our knowledge this is the first report for inducible STAT1 expression in a time- and dose-dependent manner.

## Background

The JAK-STAT pathway is known to play a pivotal role in a variety of different cytokine cascades. JAKs (Janus kinases) are associated with intracellular domains of receptors and become phosphorylated after ligand binding and aggregation of respective receptor chains. Activated JAKs phosphorylate tyrosine residues of the receptor, thereby providing docking sites for STATs (signal transducers and activators of transcription). STATs are in turn phosphorylated on tyrosine and/or serine residues, form homo- and/or heterodimers, translocate to the nucleus and activate transcription of stimulus-dependent genes [1-3]. Seven mammalian members of the STAT family are known (STAT1, 2, 3, 4, 5a, 5b, and 6) and they all share common features and structure. They have an aminoterminal DNA-binding domain, a carboxyterminal transactivation domain and SH2 domains for interaction with tyrosine phosphorylation sites [4,5]. STAT1 knockout mice show impaired response to interferon (IFN) type I and II leading to high susceptibility to viruses and other pathogens [6,7]. In addition, STAT1 is known to be involved in mechanisms like cell growth, proliferation and apoptosis [8] and phosphorylation-independent STAT1 functions have been postulated [9]. Based on the multiple functions of STAT1 identified through the generation of STAT1 knockout mice, our aim was to establish a system to study STAT1 gene function in a spatiotemporal and dose-dependent manner. In addition, we and others reported clearly reduced STAT1 protein levels in mice deficient for certain components of the IFN and TLR (toll-like receptor) signaling cascades [10,11]. Availability of STAT1 protein is critical for a number of host cell responses and limitations may well contribute to the phenotype of these mice. A system for regulated STAT1 expression would additionally provide a tool for uncoupling effects caused by STAT1 protein levels from the phenotype due to targeted deletion of the gene of interest.

During the last decade several inducible expression systems have been described and although a lot of improvements have been implemented they all have certain limitations [12]. For our purpose, high inducibility, tight regulation, suitability for *in vivo *pathogen challenges and, importantly, absence of basal transcriptional activity are the major requirements. These criteria have been described for rapamycin-regulated expression systems [13]. These systems are based on rapamycin-induced dimerization of two fusion proteins of a rapamycin binding domain each fused to a transactivation domain (TAD) or a DNA-binding domain (DBD), respectively. Both fusion proteins are expressed constitutively from a bicistronic mRNA. Upon rapamycin treatment DBD and TAD dimerize to form a functional transcription factor and in turn activate transcription from a recognition site positioned upstream of the target gene. Mutations in the rapamycin binding domain have made the use of non-immunosuppressive analogs (rapalogs) possible [14]. Rapamycin and rapalogs are orally applicable, have prolonged pharmacokinetics and can cross the blood brain barrier. The rapamycin system has been first evaluated *in vitro *[15] and in transient *in vivo *studies [16,17] and since then used with a variety of vector systems mainly for clinical development [13]. So far the system has not been described in transgenic mouse models.

We have constructed a two-vector based rapalog-inducible system for STAT1 expression and evaluated applicability in stable murine systems according to the above-mentioned requirements. Using stably transfected mouse embryonic fibroblast (MEF) cell lines we proved inducibility, tightness of expression and the biological functions of transgenically expressed STAT1 protein. We furthermore demonstrate rapalog-dependent STAT1 expression in double-transgenic embryonic stem (ES) cells, thereby providing a second, independent cell system for regulated STAT1 expression. With respect to *in vivo *applicability for transgenic mouse models, we demonstrate functionality of the individual constructs and the proof of principle in double-transgenic animals.

The inducible system described herein provides a novel tool for the analysis of time- and dose-dependent STAT1 functions and will complement the knowledge obtained from the analysis of STAT1-deficient cells and mice.

## Results

### Generation of a modified two-vector-based rapalog-inducible system for STAT1 expression

**Figure 1:**
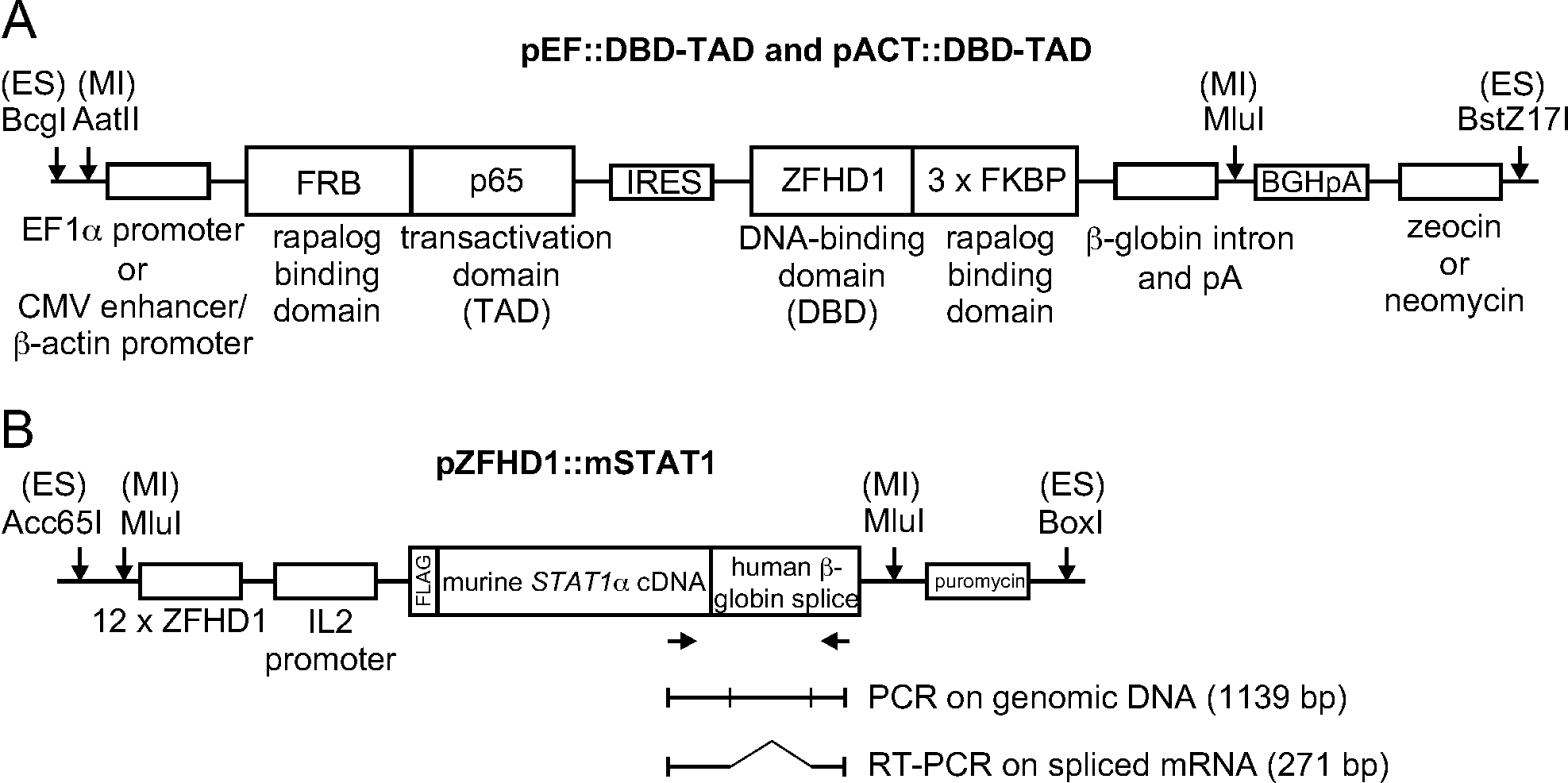
Schematic representation of the two vector types generated for inducible STAT1 expression. pEF::DBD-TAD and pACT::DBD-TAD (A) encode the two transcription factor components under the control of the EF1α promoter or the CMV enhancer/β-actin promoter. The rapalog binding domain FRB (amino acids 2021–2113 of human FRAP with the threonine at amino acid position 2098 mutated to leucine) is fused to the transactivation domain (TAD) p65 (amino acids 361–551 of human NFκB); the second rapalog binding domain 3xFKBP (three tandemly repeated copies of human FKBP12) is fused to the DNA-binding domain (DBD) ZFHD1 (a fusion of human transcription factors Zif268 and Oct-1); the two coding regions are separated by an IRES sequence from encephalomyocarditis virus and both fusion proteins contain an amino-terminal nuclear localization signal. The second vector (pZFHD1::mSTAT1) is the target vector (B) and contains 12 repeats of the ZFHD1 recognition site upstream of a minimal interleukin 2 (IL2) promoter. Murine *STAT1α *cDNA is tagged at the 5'- end with a minimal FLAG-sequence and fused at the 3'-end to the human β-globin splice sites to ensure stability of the mRNA. Zeocin, neomycin and puromycin resistance genes, respectively, were used for selection of stably transfected cells. Restriction sites utilized for isolation of the DNA fragments used for stable transfection of ES cells (ES) or microinjection into zygotes (MI) are indicated. Position of primers used for RT-PCR analysis of transgenic *STAT1 *mRNA expression are indicated (B). PCR amplicons from DNA and spliced mRNA are shown beneath. Vectors used for transfection of cells are cited in the text with "p" (e.g. pEF::DBD-TAD), constructs used for *in vivo *transgenesis without "p" (e.g. EF::DBD-TAD).

The rapalog-inducible expression system comprises of the rapalog binding components FRB (a mutated fragment from the human FKBP rapamycin-associated protein FRAP) and FKBP (FK506 binding protein) fused to a TAD (a domain from the human p65/NFκB protein) and a DBD (ZFHD1), respectively [16]. Both fusion proteins are expressed from a bicistronic mRNA (separated by an IRES sequence). Rapalog-dimerized DBD and TAD bind the ZFHD1 recognition site upstream of a minimal IL2 promoter. The DBD-TAD and the target gene components are either located on separate or within one vector construct. Our initial stable transfection experiments in murine fibroblasts indicated background expression if all components were present in one vector (data not shown) and we therefore focused on the two-vector system (Fig. 1). The original expression vector contains the transcription factor components under the control of the constitutive CMV enhancer/promoter. This promoter is frequently silenced in transgenic mouse models [18,19], thus we decided to make use of two alternative constitutive promoters [20-22]. We cloned the two transcription factor components into an expression vector under the control of the elongation factor (EF) 1α promoter [23] or the CMV enhancer/β-actin promoter (ACT) [24], repectively (pEF::DBD-TAD, pACT::DBD-TAD; Fig. 1A). The second vector was generated by insertion of the *STAT1α *cDNA into the ARGENT™ target vector (pZFHD1::mSTAT1; Fig. 1B).

### STAT1 is expressed in a dose- and time-dependent manner and is stable after removal of the dimerizer

**Figure 2:**
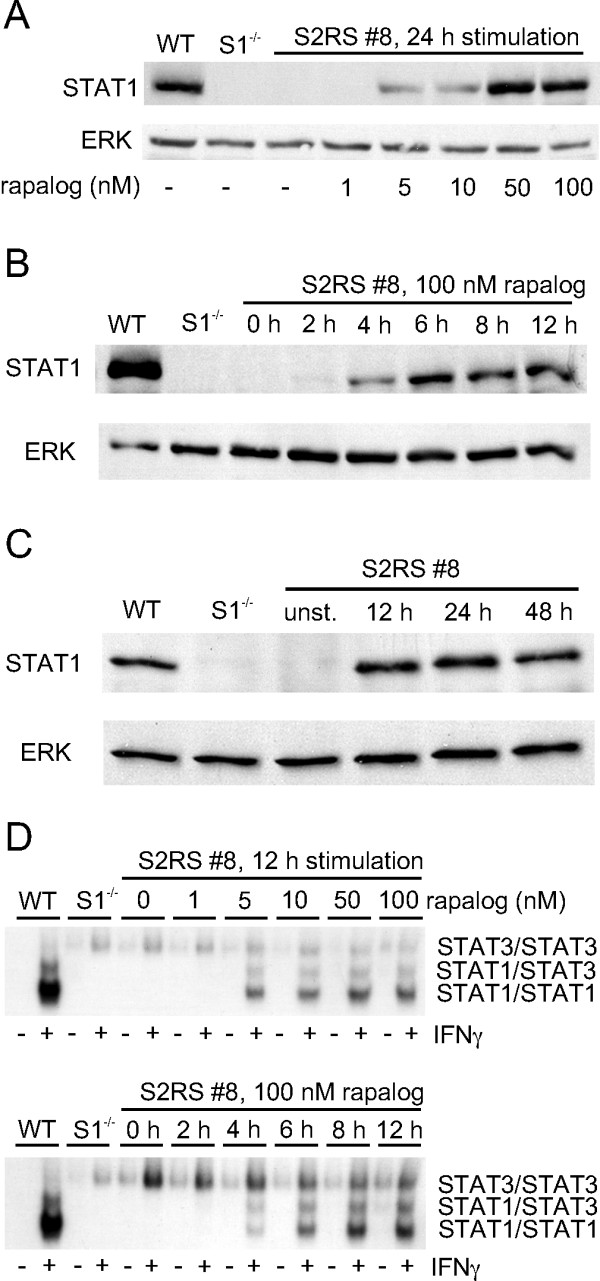
Inducible STAT1 expression in stably transfected MEF cell lines. WT, STAT1^-/- ^(S1^-/-^) and STAT1^-/- ^cells reconstituted with inducible STAT1 (S2RS #8) were treated with rapalog at the dose indicated for 24 h (A) or treated with 100 nM rapalog for the times indicated (B). To analyze STAT1 protein stability, cells were treated with 100 nM rapalog for 12 h, washed three times with PBS and further incubated without inducer. STAT1 protein expression was determined with WB analysis and loading was controlled via probing for ERK protein (A-C). Cells were treated as in A and B, stimulated with 100 U/ml IFNγ for 15 min or left untreated, lysed and 15 μg total cell extract subjected to EMSA analysis using hSIE oligonucleotides as probes (D). Similar results were obtained with other reconstituted clones.

STAT1-deficient MEF cell lines were stably transfected with the constructs pEF::DBD-TAD and pZFHD1::mSTAT1 and clones were screened for STAT1 expression. To evaluate inducibility of the system, cells were stimulated with rapalog and STAT1 expression was determined using western blot (WB) analysis. Data are shown for one representative clone (S2RS #8). STAT1 is expressed in a dose- (Fig. 2A) and time- (Fig. 2B) dependent manner upon rapalog treatment. STAT1 protein expression in the stably transfected clone S2RS #8 reaches similar levels as in wildtype (WT) cells upon 24 h treatment with 50–100 nM rapalog. STAT1 protein expression is detectable at about 2–4 h and protein levels further increase up to at least 24 h treatment.

To investigate presence of STAT1 protein after removal of dimerizer, cells were treated with rapalog for 12 h, washed with PBS and further incubated in the absence of rapalog. As shown in Fig. 2C, STAT1 protein was still detectable to similar levels 12, 24 and 48 h after removal of the extracellular dimerizer. Thus, as an important point for the *in vivo *practicability of the system, active transcription factor and/or STAT1 mRNA/protein stability is sufficient to ensure prolonged availability of STAT1 protein after elimination of extracellular dimerizer.

### IFNγ-mediated STAT1 activation is rapalog-dependent in STAT1^-/- ^fibroblasts reconstituted with inducible STAT1

Next, we tested cytokine-induced STAT1 activation upon rapalog treatment. WT, STAT1^-/- ^cells and STAT1^-/- ^cells reconstituted with inducible STAT1 (S2RS #8) were stimulated with rapalog and subsequently treated with IFNγ or left untreated. STAT1 DNA-binding activity could be detected after 12 h treatment over a range of 5–100 nM rapalog, although, consistent with the protein expression data, the level under these conditions is lower than in WT cells. DNA-binding activity was observed from 4 h treatment onwards with 100 nM rapalog (Fig. 2D) and further increased with time. In addition to the rapalog-dependent STAT1 homodimer formation also STAT1/STAT3 heterodimers could be detected in S2RS #8 cells upon IFNγ stimulation, demonstrating that transgenic STAT1 can interact normally with STAT3. In the absence of dimerizer STAT1 DNA-binding activity was not detectable, further substantiating the non-leakiness of the system. STAT1 DNA-binding activity (Fig. 2D) and STAT1 phosphorylation (data not shown) remained dependent on cytokine stimulation, which is another important determinant for the evaluation of a useful STAT1 expression system.

### Inducible STAT1 protein is a biological active transcription factor

**Figure 3:**
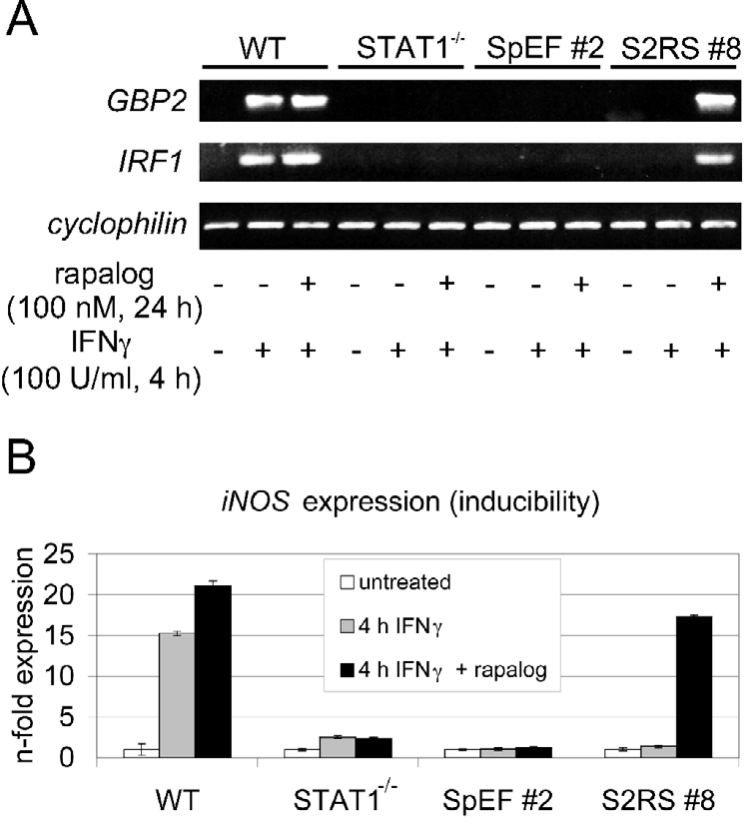
Inducible IFNγ-mediated induction of STAT1 target genes in MEF cell lines. Cells were treated with 100 nM rapalog for 24 h or left untreated and subsequently stimulated with IFNγ (100 U/ml) for 4 h or left untreated. Total RNA was used for RT-PCR analysis for *GBP2 *and *IRF1 *expression; *cyclophilin *was used as endogenous control (A). Quantitative RT-PCR was performed for *iNOS *expression, *HPRT *was used for normalization; one representative of two independent experiments is shown (B).

To examine the biological functions of the inducible STAT1 protein we stimulated cells with IFNγ and analyzed transcriptional activation of STAT1-dependent, IFNγ-inducible genes. We performed semiquantitative RT-PCR to detect *guanylate binding protein 2 (GBP2) *and *interferon regulatory factor 1 (IRF1) *mRNA (Fig. 3A) and quantitative RT-PCR (qPCR) to detect *inducible nitrite oxide synthase (iNOS) *mRNA (Fig. 3B). The reconstituted clone S2RS #8 showed transcriptional induction of all three genes upon IFNγ stimulation in the presence of rapalog. Without rapalog gene induction was not detected, again underlining the tightness of the expression system. STAT1^-/- ^cells stably expressing the transcription factor components only (SpEF #2) did not show gene induction upon IFNγ treatment in both, the absence or presence of rapalog. Quantitative analysis of *iNOS *mRNA expression revealed similar IFNγ-mediated induction in S2RS #8 and WT cells. Importantly, rapalog treatment did not influence the transcriptional activity of STAT1 in IFNγ-stimulated WT cells (Fig. 3A). The slight increase in *iNOS *mRNA expression in rapalog/IFNγ-treated WT cells (Fig. 3B) was not consistently observed. As shown for *GBP2 *and *IRF1*, *iNOS *mRNA was not induced in STAT1^-/- ^and SpEF #2 cells upon rapalog and/or IFNγ treatment. Consistent with the data for STAT1 DNA-binding activity, also gene induction remained dependent on a cytokine stimulus in STAT1-expressing cells. These results demonstrate the biological activity of the inducible STAT1 transgene at the example of three independent target genes.

### Inducible STAT1 protein can mediate antiviral protection

**Figure 4:**
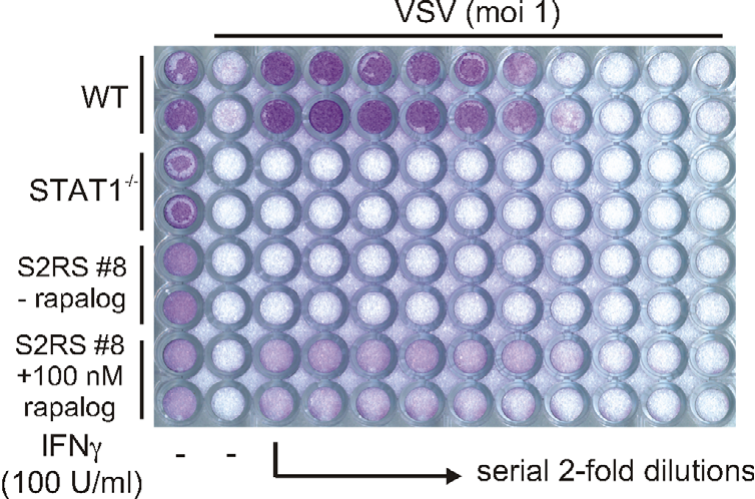
Rapalog-induced antiviral activity in IFNγ-treated MEF cell lines. Cells were treated with 100 nM rapalog for 4 h or left untreated and subsequently stimulated with serial two-fold dilutions of IFNγ (starting with 100 U/ml) for 8 h or left untreated. Cells were then challenged with VSV (moi = 1) and surviving cells were stained with crystal violet 36 h post infection.

STAT1 is crucial for the IFNγ-induced defense against vesicular stomatitis virus (VSV) infection [7]. To show that inducibly expressed STAT1 protein is also able to restore complex biological functions, we analyzed antiviral activity against VSV infection. IFNγ induced antiviral activity in rapalog-pretreated but not in untreated S2RS #8 cells (Fig. 4). The amount of IFNγ required for resistance against VSV was similar in rapalog-treated S2RS #8 and WT cells. Rapalog treatment of WT, STAT1^-/- ^and SpEF #2 cells did not influence intrinsic or IFNγ-induced resistance against VSV infection (data not shown). Hence, STAT1-dependent IFNγ responses involving multiple and complex mechanisms can be inducibly restored with this STAT1 expression system.

### Stably transfected embryonic stem (ES) cells express transgenic STAT1 protein dependent on the presence of rapalog

**Figure 5:**
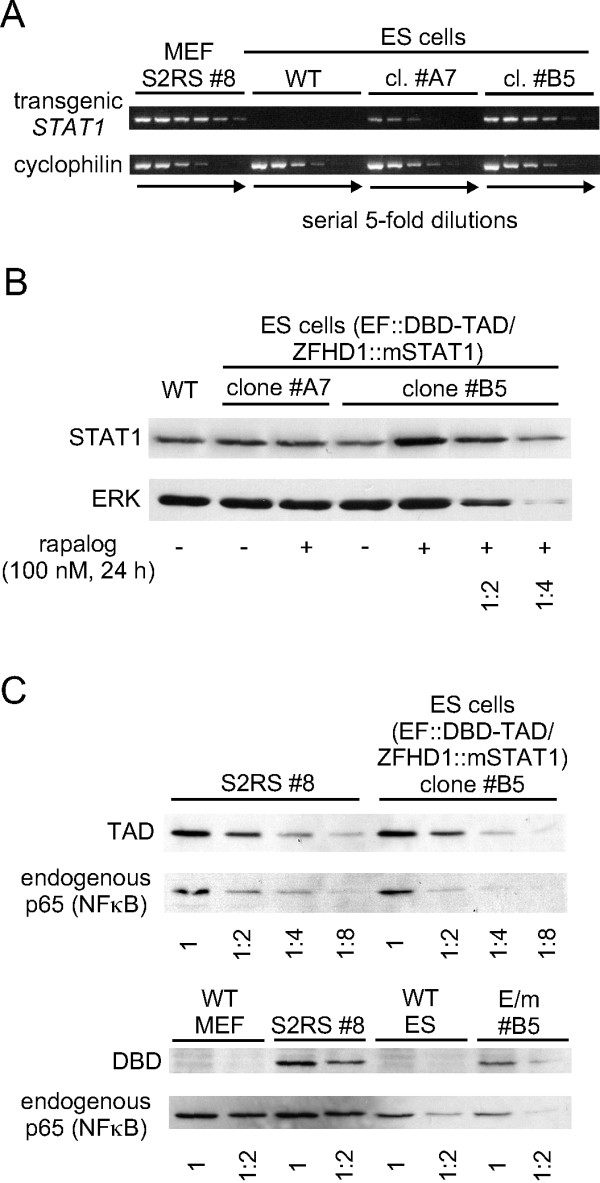
Expression analysis of inducible STAT1 and transcription factor components in double-transgenic ES cells. ES cells were stably transfected with the constructs EF::DBD-TAD and ZFHD1::mSTAT1. WT ES cells and two representative double-transgenic clones (#A7 and #B5) were treated with 100 nM rapalog or left untreated, lysed and analyzed for inducible STAT1 expression. RT-PCR was performed to detect transgenic *STAT1 *mRNA and normalized to *cyclophilin *(A). WB was performed to detect STAT1 protein as described in the legend to Fig. 2(B). Expression of the FRB-p65 fusion protein (TAD; ~48 kDa) in ES cell clone #B5 was determined by WB analysis in comparison to the S2RS #8 MEF cell line. Expression of the DBD fusion protein (~50 kDa) is shown by WB on the rapalog-binding domain FKBP12 in ES cell line #B5 and the MEF cell line S2RS #8 with respective WT cells. Endogenous p65/NFκB (65 kDa) is shown as loading control (C).

To test the rapalog-inducible STAT1 system in another independent cell system, we stably transfected ES cells with the two constructs EF::DBD-TAD and ZFHD1::mSTAT1. We identified several clones with different expression levels of rapalog-inducible STAT1. Clone #A7 expressed low transgenic *STAT1 *mRNA levels, whereas in clone #B5 rapalog-induced *STAT1 *expression was as high as in the MEF cell line S2RS #8 (Fig. 5A). As expected, no transgenic *STAT1 *mRNA was detected in WT ES cells. Expression levels of endogenous *STAT1 *mRNA in ES cell clones #A7 and #B5 was similar to WT ES cells, demonstrating that induction of transgenic *STAT1 *expression has no obvious influence on endogenous *STAT1 *expression (data not shown). Rapalog-dependent increase of STAT1 protein was clearly detectable in clone #B5, but was not observed in clone #A7 (Fig. 5B). Accordingly, the expression level of both transcription factor components TAD and DBD in clone #B5 was similar to the level in MEF cell line S2RS #8 (Fig. 5C). Increase of STAT1 protein level is difficult to analyze in ES cells since they express endogenous STAT1 and for as yet unknown reasons, we could not detect the minimal FLAG-tag fused to the transgenic STAT1. Expression of STAT1 protein upon rapalog treatment in clone #B5 clearly exceeds WT levels and, as another important point for the analysis of STAT1 function, does not lead to dramatic overexpression. Taken together, these data show that this dimerizer-inducible expression system is suitable for the use in ES cells.

### The two individual components of the expression system are functional as transgenes

**Figure 6:**
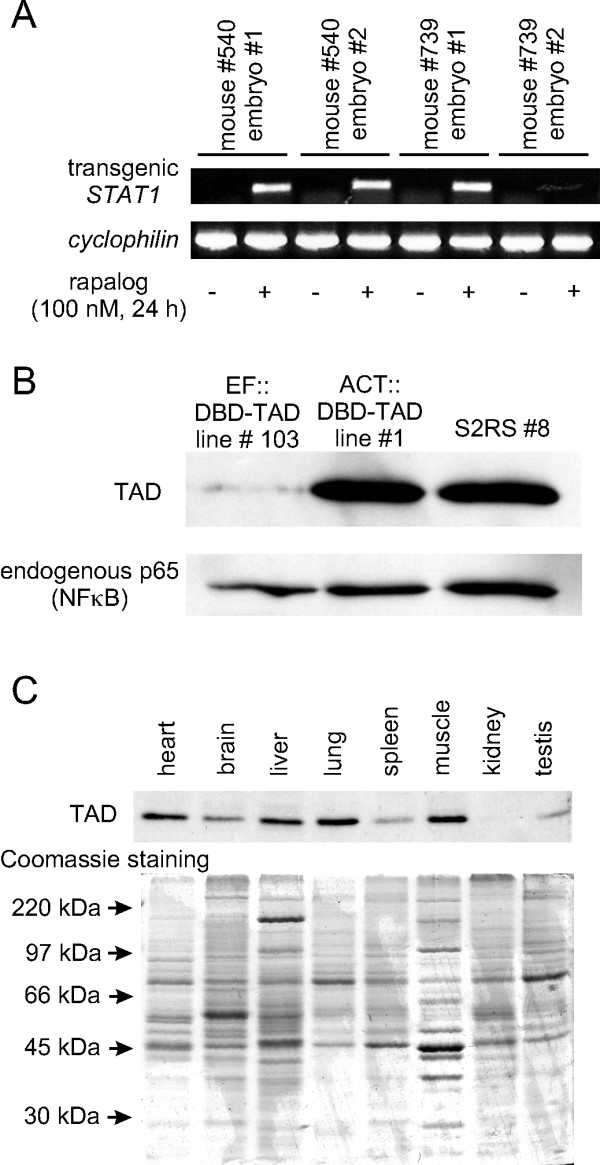
Expression analysis of single-transgenic mice for the ZFHD1::mSTAT1 or the ACT::DBD-TAD construct. Mice transgenic for the target construct ZFHD1::mSTAT1 (line #82) were crossed with non-transgenic mice. PEF were isolated from individual embryos at day 14, transfected with pEF::DBD-TAD, stimulated with 100 nM rapalog for 24 h or left untreated and subjected to RT-PCR analysis for transgenic *STAT1 *expression; *cyclophilin *was used as endogenous control (A). PEF were isolated from each embryo separately of transgenic mice (ACT::DBD-TAD, line #1 and EF::DBD-TAD, line #103), lysed and subjected to WB for analyzing expression of TAD compared to MEF line S2RS #8. Loading was controlled with p65/NFκB (B). Protein was isolated from different organs of transgenic mice and WB was performed detecting TAD subunit. As loading control a SDS-PAGE was performed and stained with Coomassie (C).

To test the applicability of the two individual vector components as transgenes respective constructs were microinjected into zygotes. Three mouse lines transgenic for the ZFHD1::mSTAT1 were obtained by DNA-microinjection and analyzed for transgene expression. Since no gene expression occurs from this construct in the absence of the transcription factor components and the dimerizer, we isolated primary embryonic fibroblasts (PEF), transiently transfected the pEF::DBD-TAD construct and analyzed rapalog-inducible transgenic *STAT1 *mRNA expression. Data are shown for PEF from four embryos of two individual mice (#540, #739) from the same transgenic mouse line (#82). PEF from at least one embryo per mouse expressed transgenic *STAT1 *mRNA (Fig. 6A). As in the stably transfected MEF cell lines, *STAT1 *expression was strictly dependent on rapalog stimulation in the transgenic situation. Similar results were obtained with a second transgenic mouse line, whereas the third line analyzed did not show any *STAT1 *expression (data not shown). These data prove the functionality of the STAT1 target construct (ZFHD1::mSTAT1) as a transgene *in vivo*. Notably, even in the presence of high amounts of the transcription factor components in the transient transfection experiment, *STAT1 *expression remained dependent on rapalog.

Mice transgenic for the transcription factor components were initially generated with the EF::DBD-TAD construct. Several transgenic mouse lines generated either via DNA-microinjection or via blastocyst injection of pre-screened ES cells showed ubiquitous but low expression of the transcription factor components as compared to the MEF cell line S2RS #8 or the ES cell clone #B5 (data not shown). Amongst nine transmitting mouse lines, five lines expressed the *DBD-TAD *mRNA, but only one of them showed detectable TAD protein expression. Expression levels of transcription factor domains were in all mice clearly lower than in the MEF cell lines and ES cells stably transfected with the same construct (Fig. 6B and data not shown). In contrast, analysis of PEF from mice generated by blastocyst injection of ES cells transgenic for the transcription factor components under the control of the CMV enhancer/β-actin promoter (ACT) revealed higher expression of TAD than in PEF derived from EF::DBD-TAD mice. TAD protein level was comparable to that in S2RS #8 cells and hence should be sufficient for inducible STAT1 expression (Fig. 6B). The transcription factor components in ACT::DBD-TAD mice were expressed in all tissues tested, although the expression level varied amongst different tissues (Fig. 6C). Heart, liver, lung and muscle showed high expression of TAD, whereas in brain, spleen and testis the expression level of transcription factor components was significantly lower and barely detectable in kidney. In addition, we observed that TAD expression also varied amongst individual mice and littermates. The majority of mice analyzed showed lower expression level of the transcription factor than the MEF cell line S2RS #8 and the ES cell clone used for the generation of these mice (data not shown).

### Double-transgenic mice (ACT::DBD-TAD × ZFHD1::mSTAT1) show inducible transgenic *STAT1 *mRNA upon rapalog stimulation

**Figure 7:**
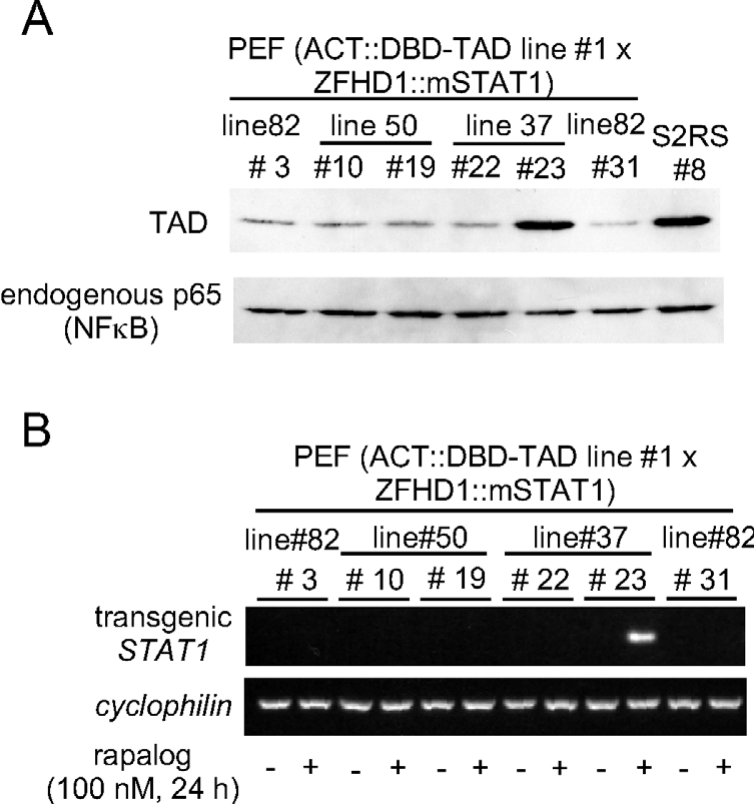
Expression of transcription factor components and transgenic STAT1 in double-transgenic mice. PEF were isolated from double-transgenic mice (ACT::DBD-TAD line #1 × ZFHD1::mSTAT1) and stimulated with rapalog (100 nM) for 24 h or left untreated (A, B). Cells were lysed, and WB was performed to detect TAD. p65/NFκB was used as endogenous control (A). RNA was isolated and RT-PCR performed to detect transgenic *STAT1 *mRNA; *cyclophilin *was used as endogenous control (B).

To investigate whether the amount of transcription factor components in ACT::DBD-TAD mice (line #1) is sufficient to induce transgenic STAT1 *in vivo*, these mice were crossed with three different ZFHD1::mSTAT1 mouse lines (#37, #50, #82). PEF were isolated from single embryos, genotyped and double-transgenic clones were first screened for expression of the transcription factor components. PEF from one out of six embryos showed high expression levels of the transcription factor components (Fig. 7A). In PEF #23 TAD expression was similar to the MEF cell line S2RS #8, whereas PEF derived from all other embryos showed clearly lower expression levels (Fig. 7A). Next, we stimulated these double-transgenic PEF with rapalog and analyzed transgenic *STAT1 *mRNA expression by RT-PCR. Basal expression of transgenic *STAT1 *was not seen in PEF from any of the embryos tested, thereby proving the tightness of the system also *in vivo*. Transgenic *STAT1 *mRNA could be induced upon rapalog stimulation in PEF #23, which had high TAD expression levels (Fig. 7B), but not in cells with low TAD expression. Although *STAT1 *mRNA was clearly induced, we could not detect STAT1 protein against the background of endogenous STAT1 (data not shown). However, this set of experiments shows that transgenic *STAT1 *can be induced in PEF derived from double-transgenic mice and that the dimerizer-based system is in principle functional in transgenic mouse models.

## Discussion

In this study we established a rapalog-inducible expression system for STAT1. Using STAT1^-/- ^MEF cell lines stably reconstituted with the inducible STAT1 expression system, we show that STAT1 protein is tightly controllable: in the absence of inducer no expression is detectable and the amount of expression is dependent on the dose of dimerizer applied. Although expression levels varied, as expected, amongst different clones, a reconstituted cell line with STAT1 levels up to WT cells could be obtained. Induction occurs rapidly, increases over time and STAT1 protein is stable after removal of dimerizer for at least up to 48 h (Fig. 2). Inducible STAT1 protein is biologically active and can restore the IFNγ response defects in STAT1^-/- ^cells. No effects on IFNγ responses were observed in cells expressing the DBD-TAD domains alone (Fig. 3 and 4). We generated stably transfected ES cells and proved the functionality of the STAT1 expression system in an independent cell type and in a STAT1^+/+ ^background (Fig. 5). Recently, inducible expression systems have been described for the use in ES cells, mainly based on the tetracycline system [25]. To our knowledge this is the first report, demonstrating dimerizer-based inducible gene expression in ES cells.

We present several attempts to apply this system in transgenic mouse models and prove the principle of *in vivo *applicability. We analyzed the functionality of each of the constructs of the two vector-based system in single-transgenic mice (ZFHD1::mSTAT1 and EF::DBD-TAD, respectively) and show that inducible expression can be achieved by transient transfection with the respective second construct. Expression of STAT1 remained strictly dependent on the dimerizer even in the presence of access of either of the two constructs (Fig. 6A and data not shown). Transgenic mice for the transcription factor components under the control of the EF1α promoter (EF::DBD-TAD) showed ubiquitous but low level of expression. PEF from double-transgenic mice obtained by crossing ZFHD1::mSTAT with EF::DBD-TAD did not show any STAT1 induction upon rapalog treatment. Lack of STAT1 induction in these mice is most likely due to the low expression of the transcription factor components, since direct correlation of DBD-TAD expression and target gene expression has been reported before [13]. In contrast, mice transgenic for the transcription factor components under the control of the CMV enhancer/β-actin promoter [24] (ACT::DBD-TAD) showed higher expression levels of the transcription factor components. At least in PEF from one embryo tested, expression levels were similar to the levels obtained in the stable transfected MEF and ES cell clones that do express sufficient amounts for STAT1 protein induction (Fig. 6B). Higher *in vivo *expression correlated with higher expression levels in ES cells *in vitro *(data not shown). Unfortunately, the expression pattern of DBD-TAD in these mice was variegated between organs and even between full siblings (Fig. 6C and data not shown), a phenomenon that has been widely described for transgenic animals [26,27]. Upon crossing these mice with single-transgenic ZFHD1::mSTAT1 mice, PEF from one out of six embryos showed inducible *STAT1 *expression. *STAT1 *expression was again dependent on rapalog stimulation proving the non-leakiness of the system *in vivo*. *STAT1 *inducibility correlated with high expression of DBD-TAD, further underlining that sufficient DBD-TAD expression is one of the limiting factors of the system (Fig. 7). However, we could not detect transgenic STAT1 protein against the background of endogenous STAT1 even in the presence of high amounts of transcription factor components (data not shown). One possible explanation is, that transgene silencing effects [28] also occur in ZFHD1::mSTAT1 mice and that the accessibility of the target promoter is also gene locus dependent. Since transient transfection of DBD-TAD into cells derived from these mice does lead to sufficient STAT1 induction (Fig. 6A and data not shown) it is most likely a combinatorial effect of the integration loci of both constructs. One possible solution would be to screen a large number of independent single- and double-transgenic animals for stable and sufficient DBD-TAD expression and STAT1 inducibility. Since this is a very time- and animal-intensive approach, future studies will focus on homologous integration of both, DBD-TAD and ZFHD1::mSTAT1 separately into ES cells. Single copy integration of the transgenes into established loci in the mouse genome should avoid any silencing mechanisms and ensure defined and stable expression levels *in vivo *[29]. Variegated DBD-TAD expression amongst individuals/littermates and the crucial role of DBD-TAD levels for STAT1 induction makes *in vivo *rapalog treatment experiments with ZFHD1::mSTAT1 × ACT::DBD-TAD mice currently inpracticable. Additional complexity in variability might come from interindividual differences in pharmacokinetics and/or intracellular bioavailability of the transcription factor components and rapalog. At this stage this cannot formally be excluded. However, *in vivo *functionality of rapamycin-derivatives as synthetic inducers of biological functions and of the transcription factor components in various cellular environments has been demonstrated [30-32]. In contrast to other synthetic inducible expression systems, i.e. ecdysone or tetracycline [12], the TAD used here might undergo post-translational modifications. Indeed, in preliminary experiments we have observed conditional phosphorylation of TAD at one specific site paralleling the modification of the endogenous p65/NFκB (data not shown). *In vitro*, in MEF cell lines and ES cells, phosphorylation-independent TAD activity leading to rapalog-induced STAT1 expression has been demonstrated (Fig. 2 and 5). Further studies are required to address the dependence of TAD activity on the status of the endogenous p65/NFκB regulation with respect to additional known phosphorylation sites and different cellular contexts. However, variegated expression of the DBD-TAD components is currently the crucial point for *in vivo *functionality of this system. Therefore, this makes it to our opinion worthwhile to solve the issue of obvious chromatin positioning effects on the transgenes.

For the use of inducible expression systems no or minimal off-target effects are preferable. As with other systems, possible side-effects cannot be excluded and have to be assessed for each specific application. Here, no off-target effects concerning the dimerized transcription factor components or rapalog treatment alone could be observed in two independent biological assay conditions (Fig. 3 and 4).

In summary, the rapalog-inducible STAT1 expression system established in this report provides a novel tool to study dose- and time-dependent STAT1 functions in fibroblasts and embryonic stem cells. Our attempts to introduce the system into transgenic mice gave proof of principle functionality and future directives for improvements.

## Conclusion

Inducible temporal gene expression systems are indispensable tools for fine-tuned analysis of gene functions. All reported systems have some limitations [33]. Here we report the application of a novel rapalog-inducible STAT1 expression system *in vitro *and *in vivo*. STAT1 is a transcriptional activator central for the signaling of a variety of cytokines and growth factors. STAT1 functions range from immune regulation to tumorigenesis and timely availability of STAT1 protein is thought to be crucial for (ab-)normal cellular function. We show that rapalog-induced STAT1 expression *in vitro *is highly inducible and tightly controllable demonstrating the potential of this inducible system. Using reconstitution experiments we show that rapalog-induced STAT1 fulfills all main STAT1 functions and thus is a novel tool to unravel STAT1 functions in a time- and dose-dependent manner.

We show that rapalog-inducible STAT1 expression can be achieved *in vivo*, although STAT1 induction is impaired by transgene silencing effects in transgenic mice harbouring randomly integrated gene constructs. These effects can be avoided by stable integration of single copies of each expression vector into established loci in the mouse genome. An inducible STAT1 expression system *in vivo *could identify STAT1-independent gene functions in several knockout mice with reduced STAT1 level. A rapalog-inducible expression system would also be a powerful alternative to existing inducible expression systems *in vivo*.

## Methods

### Expression vectors

Transcription factor domains of the vector pC_4_N_2_-R_H_S/ZF3 (ARIAD Pharmaceuticals, Inc.) were cloned into the expression vector pEF-zeo (kindly provided by Pavel Kovarik, Max F. Perutz Laboratories, University of Vienna, Austria) [23,34], containing the EF1α promoter and a zeocin resistance gene. A second vector containing the transcription factor domains under the control of a CMV enhancer/β-actin promoter [24] was cloned into pKO-sc920 (Lexicon Genetics) containing a neomycin resistance cassette. *STAT1α *cDNA (kindly provided by Chris Schindler, Columbia University, New York) [35] was N-terminal tagged with a FLAG (MDYKDED, [36]) and a human β-globin splice (GenBank accession no. U01317, nt 62553–64242) was added to the 3'-end. The modified *STAT1α *cDNA was cloned into the target vector pZ_12_I-PL2 (ARIAD Pharmaceuticals, Inc.) and a puromycin resistance gene (from the vector pKOSelect-PuroV810; Lexicon Genetics) was inserted downstream of the *STAT1α *cDNA.

### Reagents

Purified, recombinant murine IFNγ was obtained from Calbiochem. Antibodies were purchased from Santa Cruz (STAT1α p91 (M-23)), Upstate (Anti-NF-κB p65, CT), Transduction Laboratories (pan ERK) and Biovision (FKBP12) respectively, and used at dilutions of 1:250 (FKBP12) or 1:1000 (all other antibodies). HRP-coupled anti-rabbit antiserum and anti-mouse IgG were used from Sigma Aldrich and Amersham Biosciences, respectively (1:2000 dilutions). Rapalog AP21967 [14] was obtained from ARIAD Pharmaceuticals, Inc. and used at concentrations and times indicated. Coomassie staining was performed with Bio-Safe Coomassie Stain (BioRad).

### Cell culture

Primary embryonic fibroblasts (PEF) were isolated as described [37]. Wildtype (WT) embryonic fibroblast cell lines (MEF) were generated by spontaneous immortalization. The STAT1-deficient cell line was kindly provided by David Levy (New York University School of Medicine, New York) [6]. Fibroblasts were cultured in DMEM supplemented with 10 % FCS, 100 μg/ml penicillin, 100 U/ml streptomycin, and 1 mM L-glutamine (Invitrogen). E14.1 embryonic stem (ES) cells (129/OlaHsd) were cultured as described [11]. Bone marrow-derived macrophages were isolated and grown as described [38].

### Cell transfection, selection of transgenic clones

Transfections of immortalized fibroblasts with both constructs (pEF::DBD-TAD and pZFHD1::mSTAT1) were performed with TransFast™ reagent (Promega Corporation), all other transfections with Nucleofector technology (Amaxa Inc.) according to the manufacturer's instructions. For selection of stably transfected fibroblasts 5 μg/ml puromycin (Sigma Aldrich) and/or 500 μg/ml zeocin (Invitrogen) was used. Stably transfected ES cells were selected using 40 μg/ml zeocin, 0.4 μg/ml puromycin or 0.2 mg/ml G418 (Invitrogen).

### Generation of transgenic mice

Transgenic mice were either generated by microinjection of each of the vectors separately into zygotes or by blastocyst injection of transgenic ES cells. DNA fragments for microinjection (MI) or transfection of ES cells (ES) for blastocyst injection were isolated by digestion at sites indicated (Fig. 1). All animal experiments were conducted in accordance with protocols approved by the Austrian laws and European directives.

### Western Blot (WB) and Electrophoretic Mobility Shift Assay (EMSA)

1 – 5 × 10^5 ^cells were lysed and 10–15 μg protein used for WB and EMSA analysis as previously described [11,39,40].

### RT-PCR

Total RNA was isolated with Trizol Reagent (Invitrogen). cDNA was synthesized using Oligo(dT)_12–18 _primer and SuperScript™ II Reverse Transcriptase (Invitrogen). The following primers were used for RT-PCR: *cyclophilin *5'-GAC GCC ACT GTC GCT TTT CG-3' and 5'-CAG GAC ATT GCG AGC AGA TGG-3', *IRF1 *5'-CAG AGG AAA GAG AGA AAG TCC-3' and 5'-CAC ACG GTG ACA GTG CTG G-3', *GBP2 *5'-TGC TAA ACT TCG GGA ACA GG-3' and 5'-GAG CTT GGC AGA GAG GTT TG-3, transgenic *STAT1 *5'-TCT GTG TCT GAA GTC CAC C-3' and 5'-CAA GAA AGC GAG CTT AGT GAT AC-3' and *DBD-TAD *(transcription factor components) 5'-AGG CTG GGA AGA AGG GGT TGC-3' and 5'-GCC AGA AGT CAG ATG CTC AAG-3.

### Quantitative RT-PCR (qPCR)

Total RNA was isolated with Trizol Reagent (Invitrogen), DNA contaminations were removed by treatment with RQ1 RNase free DNase (Promega Corporation) and cDNA was transcribed using the iScript cDNA Synthesis Kit (BioRad). qPCR was performed as described [41] except that 1U Firepol (Solis BioDyne) with the according buffer B per reaction was used and the thermal cycling conditions were the following: 15 min at 95°C followed by 40 cycles of 15 sec at 95°C and 55 sec at 60°C. Primers used for *iNOS *qPCR: 5' TGG TCC GCA AGA GAG TGC T 3' (iNOS-fwd) and 5' CCT CAT TGG CCA GCT GCT T 3' (iNOS-rev). The oligonucleotide used as probe (5' FAM-CCC GGC AAA CCC AAG GTC TAC GTT C-BHQ1 3') was labeled at the 5'-end with the reporter dye 6- carboxyfluorescein (FAM) and at the 3'-end with the quencher dye blackhole quencher1 (BHQ1). The qRT-PCR assays for *iNOS *were submitted to the Real-Time Primer and Probe Database (RTPrimerDB identification no. 3483) [42]. *HPRT *was used as endogenous control (RTPrimerDB identification no. 3600) and the calculation of qPCRs was performed as described [41]; data were calibrated to the untreated state of the respective genotype.

### Antiviral Assays

Antiviral assays with vesicular stomatitis virus (VSV, Indiana strain) were performed as described [7].

## Authors' contributions

NRL performed cloning of the vectors, *in vitro *and *in vivo *analysis and helped in the draft of the manuscript. BS drafted the manuscript and participated in the design and performance of *in vitro *experiments. MB performed WBs of *in vitro *experiments and genotyped the mice. RP carried out the qRT-PCR analysis. TK generated transgenic mice and organized the breedings. TR organized the mouse unit. MM and MK conducted the concept and design of this work and critically revised the manuscript. All authors read and approved the final manuscript.
